# Bleeding Revived

**Published:** 1889-01-19

**Authors:** 


### Within the Wards.

### "BLEEDING" REVIVED.

Many persons think whatever is newest is best. This is particularly the case in medical practice. Some men labour with all their might to "collect all the new things," and to be "abreast of the times." That is well, so far as it goes. The art of medicine cannot claim an ideal perfection, and he who would be thoroughly efficient must be constantly learning. But it is possible to learn many things from the past as well as from the present. Some of the discarded methods of former times are better than some of the practices now in use. "Bleeding" has gone almost entirely out of fashion. Yet it is difficult to believe that bleeding could ever have attained the reputation it once enjoyed if there had been absolutely nothing in it. There must have been a large number of cases in most men's practice in which bleeding had proved itself of evident value. We refuse to believe otherwise. Nothing but proved success could ever have established the operation in such universal and unquestioned repute. A desperate case which was treated at a small county hospital a few months ago well illustrates the value of "bleeding" in certain emergencies. A young collier, twenty-one years of age, was taken to the hospital with his face, body, and limbs so swollen with dropsy as to make him scarcely recognisable. He was driven to the hospital in a cab, and by the time he was put to bed he was in a semi-comatose condition, and apparently dying. His illness was acute inflammation of the kidneys, and he had caught it by standing in water when at work in the coal-pit. His breathing when taken into the ward was stertorous, he could hardly be roused to answer questions, and violent epileptiform convulsions came on at intervals of two hours. After the last of these fits the breathing ceased, and the patient was thought to be dead. Means were taken to restore animation, and they proved successful. The patient began to breathe again and the pulse to beat. All who were present, however, and particularly the doctor, who best appreciated the danger, thought that one more convulsive fit would certainly close the scene. At this crisis the physician, who probably knew no modern remedy on which he could rely with certainty, resolved to resort to bleeding. He could not do any harm; he might do good. The operation was accordingly performed, and more than twelve ounces of blood were taken from the arm. The results were evident immediately. The breathing became easier and quieter; the power of speech gradually returned; in three hours the patient was fully and perfectly conscious. The fits did not return. Other measures were now adopted to restore the activity of the kidneys, and in two days these were completely successful. Then the dropsy rapidly disappeared, strength began to return, and the patient was soon convalescent. He left the hospital five weeks after entering it in good health. There is practically no doubt that the timely bleeding saved the patient's life. The main lesson to be learnt is this, that no medical man should be content to be a mere imitator of the medical fashions of his time, but should diligently seek light and guidance from every quarter; and not only so, but he should fearlessly rely upon his own experienced judgment rather than follow a merely routine treatment in cases of critical emergency.

